# The Development of an Internet-Based Treatment for Problem Gamblers and Concerned Significant Others: A Pilot Randomized Controlled Trial

**DOI:** 10.1007/s10899-017-9704-4

**Published:** 2017-07-11

**Authors:** Anders Nilsson, Kristoffer Magnusson, Per Carlbring, Gerhard Andersson, Clara Hellner Gumpert

**Affiliations:** 1 Department of Clinical Neuroscience, Stockholm Center for Psychiatry Research and Education, Karolinska Institutet, Stockholm, Sweden; 2 Department of Psychology, Stockholm University, Stockholm, Sweden; 3 Department of Behavioral Sciences and Learning, Linköping University, Linköping, Sweden

**Keywords:** Gambling, Cognitive behavioral therapy, Behavioral couples therapy, Internet-based interventions, Feasibility

## Abstract

Problem gambling creates significant harm for the gambler and for concerned significant others (CSOs). While several studies have investigated the effects of individual cognitive behavioral therapy (CBT) for problem gambling, less is known about the effects of involving CSOs in treatment. Behavioral couples therapy (BCT) has shown promising results when working with substance use disorders by involving both the user and a CSO. This pilot study investigated BCT for problem gambling, as well as the feasibility of performing a larger scale randomized controlled trial. 36 participants, 18 gamblers and 18 CSOs, were randomized to either BCT or individual CBT for the gambler. Both interventions were Internet-delivered self-help interventions with therapist support. Both groups of gamblers improved on all outcome measures, but there were no differences between the groups. The CSOs in the BCT group lowered their scores on anxiety and depression more than the CSOs of those randomized to the individual CBT group did. The implications of the results and the feasibility of the trial are discussed.

## Introduction

Approximately 2.3% of the Swedish population aged 16–85 years are considered to be either problem gamblers or moderate-risk gamblers (Swedish National Institute of Public Health 2010), as measured by the Problem Gambling Severity Index (PGSI) (Ferris and Wynne 2001). Problem gambling is associated with significant harm for the gambler such as economic difficulties, psychological distress, comorbid substance abuse, suicidality and physical health problems (Langham et al. 2016; Moghaddam et al. 2015; Petry 2009; Swedish National Institute of Public Health 2010).

A Swedish study estimated that 18% of the general population in Sweden could be considered as concerned significant others (CSOs) of someone who is or has been a problem gambler (Svensson et al. 2013). Problem gambling produces significant harm and distress for the CSOs of the problem gambler. Problem gambling is also known to cause financial problems for the CSOs of problem gamblers, as well as psychological and physical ill health (Downs and Woolrych 2010; Kalischuk et al. 2006; Patford 2009). Many CSOs report that their relationship with the problem gambler is severely affected, and they also describe disturbed relationships with other family members and friends (Shaw et al. 2007; Wenzel et al. 2008).

Various treatment programs have been evaluated for problem gambling and many of the successful treatment approaches are based on knowledge gained within the framework of treating substance-use disorders (Ferentzy and Turner 2013). This seems logical given the overlap in symptoms between problem gambling and substance-use disorders, as well as a possible parallel biological dysfunction and a substantial degree of comorbidity (Frascella et al. 2010; Goudriaan et al. 2006; Lorains et al. 2011; Petry 2009). In a Cochrane review (Cowlishaw et al. 2012) of psychological treatments for those with a gambling disorder, the authors found support for the efficacy of *cognitive behavior therapy* (CBT). Based on the results from seven randomized controlled trials (RCTs), the authors concluded that CBT reduced gambling behaviors (Gambling symptom severity; Cohen’s *d* pre–post-test: −1.82; 95% CI −2.61 to −1.02) and depression and anxiety symptoms compared to a control condition. Two other meta-analyses have reached similar conclusions (Gooding and Tarrier 2009; Pallesen et al. 2005) and two RCTs finding support for CBT interventions have been performed in a Swedish context (Carlbring et al. 2010, 2012).

Despite the accumulation of negative consequences associated with problem gambling, and despite the promising results of CBT for problem gambling, a mere 5–12% of problem gamblers ever seek treatment (Slutske 2006; Swedish National Institute of Public Health 2010). This has generally been attributed to stigma, a lack of accessibility to treatment, and/or an unwillingness to admit to the problem and a desire to handle problems oneself (Bellringer et al. 2008; Clarke et al. 2007; Suurvali et al. 2009). Among those who do seek treatment, adherence is low and attrition rates are high; a systematic review found that, on average, 42% of participants drop out of psychological interventions for problem gambling (Melville et al. 2007). However, including CSOs in the treatment increases gambler retention and merely having a CSO seems to increase the odds of successful treatment (Ingle et al. 2008; Kourgiantakis et al. 2013). Furthermore, concerns of CSOs have been identified as one of the main reasons for problem gamblers entering treatment (Hing et al. 2012; Ingle et al. 2008; Tepperman et al. 2006). In addition, the CSOs may have a limited understanding of—and may be less aware of—the full extent of the gambling problem (Tepperman et al. 2006), a situation which could unintentionally enable further gambling (Patford 2009).

Data from the Swedish National Gambling Helpline shows that roughly half of the helpline contacts are with the CSOs of gamblers, which indicates that among CSOs, there is an unmet need for support (Stockholm Centre for Psychiatric Research 2016). Parents and partners make up the majority of CSO contacts at roughly 30% each, while friends, siblings and relatives make up approximately 10% each. Children, including adult children of gamblers, make up less than 5% of the helpline contacts. However, research on interventions for problem gambling involving CSOs is scarce. A study of CBT group treatment where family members were invited to participate in the treatment found that CSO involvement was associated with a higher relapse rate (Jimenez-Murcia et al. 2015) and the authors recommended that CSOs and gamblers should be given separate interventions. Another pilot study involving 18 couples studied the impact of *congruence couple therapy*, involving both the gambler and a partner in couple therapy. Contrary to the study by Jimenez-Murcia et al., the latter showed significant changes in gambling symptoms and in psychological distress for the CSO (Lee and Awosoga 2015).

A handful of studies have studied interventions involving solely the CSOs of problem gamblers. Three studies have investigated *community reinforcement and family training (CRAFT)* for gambling (Hodgins et al. 2007; Makarchuk et al. 2002; Nayoski and Hodgins 2016). CRAFT was originally aimed at working with the CSOs of people with alcohol and substance problems, but has been adapted to suit the CSOs of problem gamblers. The major aim is to get “treatment-refusing” gamblers into treatment, and while this has been a successful intervention for substance abuse (Roozen et al. 2010), CRAFT for gambling has so far failed to increase treatment engagement, but has produced significant results regarding the number of days gambled (among the gamblers) and also program satisfaction for the CSOs. Another study investigating the impact of coping skills training for CSOs found significant reductions in the symptoms of depression and anxiety among CSOs, but there were no changes in treatment entry or gambling (Rychtarik and McGillicuddy 2006).

CSO involvement in clinical trials targeting alcohol and substance abuse has been somewhat better studied. One treatment approach that has been successful in treating the person with an addiction as well as involving the spouse in the treatment is *behavioural couples therapy* (BCT) (Meis et al. 2012). BCT resembles CBT approaches to substance abuse, such as traditional CBT and CRAFT, and incorporates CBT techniques targeting substance abuse such as functional analysis, relapse prevention and behavioral activation with interventions targeting relationship functioning (O’Farrell and Fals-Stewart 2006). BCT involves both the user and a CSO and has two main goals: (1) to build support for abstinence and (2) to improve relationship functioning. The hypothesized mechanism of change is that improved relationship functioning will promote relationship behaviors that are conducive to abstinence (O’Farrell and Fals-Stewart 2006). A meta-analysis of 12 BCT RCTs, of which 8 targeted alcohol problems and 4 targeted other substances, showed better outcomes for BCT than for individual-based treatments, with a mean overall between-group effect size of Cohen’s *d* = 0.44 in favour of BCT (Powers et al. 2008). BCT has been tested for different types of relationships (e.g. heterosexual couples, same-sex couples and the parent–child relationship) and for different types of substances (e.g. alcohol, illegal drugs and methadone). BCT is without doubt the therapy involving significant others aimed at addiction with the most robust research support to date (Fletcher 2013). As mentioned above, there is already some support for the notion that CSO involvement in problem gambling treatment produces better outcomes for the gambler in terms of gambling and for CSOs in terms of psychological distress, even though the results are somewhat ambiguous. It thus seems appropriate to investigate the potential effects of BCT on problem gambling.

According to the Swedish Gambling Authority, more than half (55%) of the gamblers in Sweden play online (Swedish Gambling Authority 2015). Figures from the Swedish National Gambling Helpline reveal that the three most common problem games among gamblers contacting the helpline were Internet-related: Internet casinos, Internet betting and Internet poker (Stockholm Centre for Psychiatric Research 2016). Not only are the gambling problems increasingly Internet-associated, but a growing number of contacts to the helpline are made through chat or e-mail. Internet-delivered treatments have been proposed as potential treatment alternatives for problem gambling, in part because they are flexible, anonymous and available nationwide, and thus could lower barriers to treatment. Carlbring et al. (2012) tested Internet-delivered CBT in Sweden and found significant reductions in gambling problems as well as in comorbid disorders. The Swedish studies have also been replicated in a Finnish context (Castren et al. 2013). Several other studies also suggest that Internet-delivered interventions could be viable treatment options (Canale et al. 2016; Myrseth et al. 2013; Rodda et al. 2013). Given the above-mentioned issues regarding the reluctance among problem gamblers to seek and remain in regular face-to-face treatment, in combination with the lack of studies investigating the role of CSOs in problem gambling treatment, we found reason to further develop and study treatment approaches for problem gambling.

### Aim

The primary aim with the current study was to investigate whether the involvement of CSOs in treatment would affect treatment response among problem gamblers in an Internet-delivered pilot study comparing two conditions: BCT involving both the gambler and a CSO versus CBT for the gambler only. Further, a secondary aim of this pilot study was to investigate the feasibility of the program before conducting a full-scale RCT.

## Recruitment

The study included 18 pairs (36 individuals) recruited from the Swedish National Gambling Helpline via an online advertisement and through health care professionals who were informed about the study. The gamblers met the criteria for problem gambling per the PGSI, defined as a total score ≥ 8. The CSO had to be a partner, a family member or a friend of the gambler, and they had to have known each other for at least 3 months. Furthermore, the CSO could not meet the PGSI criteria for ongoing gambling problems, and neither the gambler nor the CSO could display symptoms of severe psychiatric disorders such as psychotic or bipolar disorders that were judged to require further treatment. Participants were required to live in Sweden, be able to understand and write Swedish and be at least 18 years old.

## Procedure

**Fig. 1:**
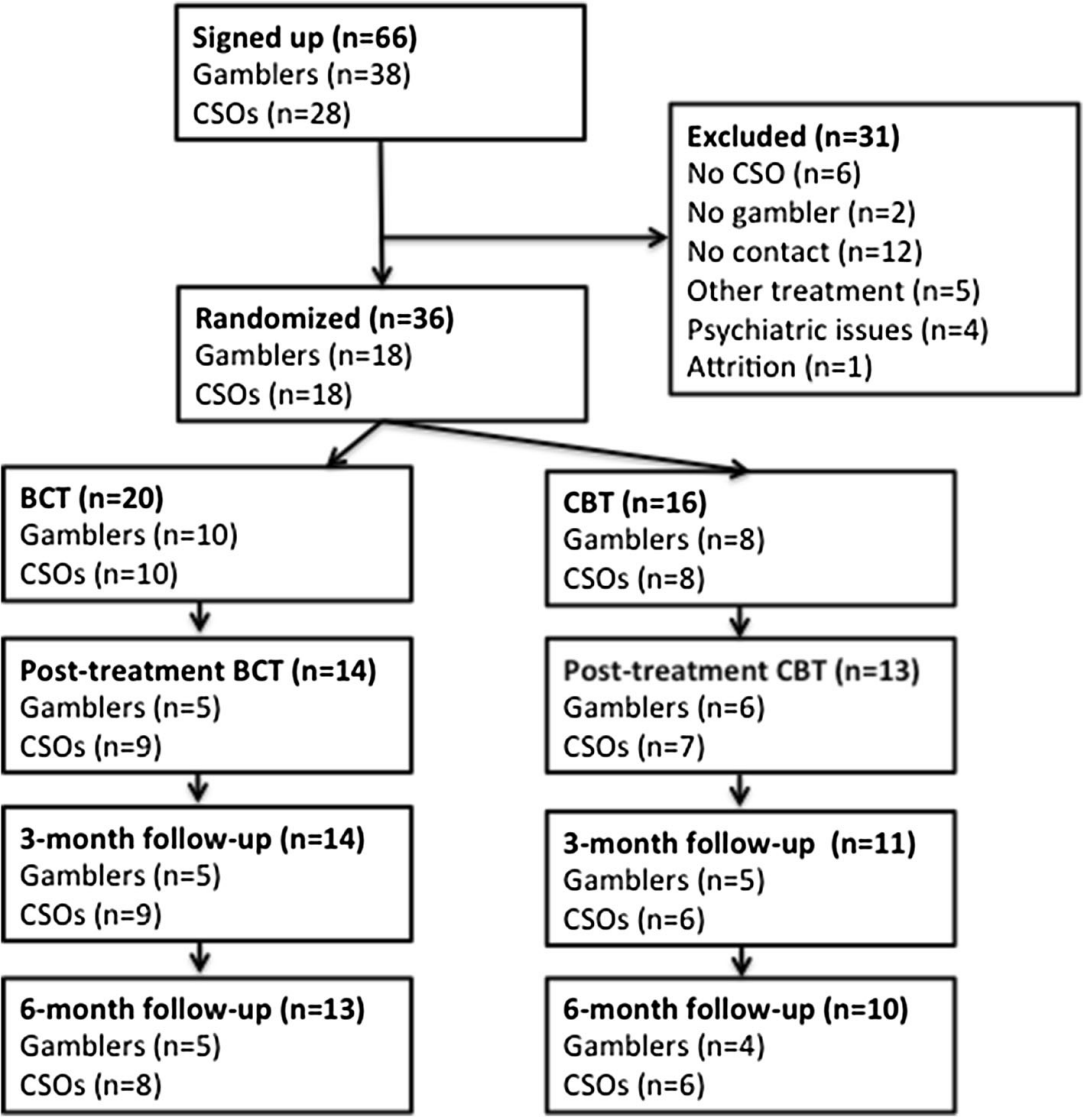
Participant flow

The participants signed up for the study through the study website (www.spelfri.se), where they filled out the online screening form. A total of 73 individuals commenced filling out the screening form while admission was open between March 26th 2015 and August 27th 2015. 45 identified themselves as gamblers and 28 as CSOs. Seven individuals did not complete the full screening procedure, all of who were gamblers. See Fig. 1 for participant flow. After signing up online, the prospective participants were contacted by telephone by a therapist. The telephone calls functioned as a complement to ensure that the participants fulfilled the eligibility criteria, that they understood the aim and design of the study, as well as to clarify any ambiguity in their replies to the screening instruments. Participants had the possibility to ask questions to better understand the purpose and the design of the study.

When the eligibility assessment was completed, each pair was randomized to one of the two study arms: CBT or BCT. Since the gambler and the CSO participated in the study together, they were randomized as one unit. The allocation sequence was generated using a true random number generator (“random.org”). A research assistant who was independent from the study performed the treatment allocation. In one case, participants in the same unit were accidently randomized separately: the gambler was randomized to the CBT group and the CSO to the BCT group. It was decided to assign them to the BCT group since the CSO had already been offered the treatment included in the BCT group, hence the uneven group sizes.

## Measures

All baseline and outcome measures were collected through various self-report measures, administered online through the treatment platform (Vlaescu et al. 2016) and filled out by both the gambler and the CSO. The gambler and the CSO were not able to gain access to the information provided by the other participant.

### Baseline Measures

Data on the following demographic characteristics was collected at baseline: age, gender, most problematic form of game, gambling-related debt and the number of years with a gambling problem. Furthermore, the data for all the outcome measures was collected at baseline (see below).

### Primary Outcomes

The primary outcome measure was the National Opinion Research Center Screen for Gambling Problems (NODS) (Wickwire Jr et al. 2008). The NODS is a 17-item self-report questionnaire with a maximum score of 10, it has been found to have acceptable psychometric properties and it corresponds to the diagnostic definition of pathological gambling in the *Diagnostic and Statistical Manual of Mental Disorders, 4th Edition* (DSM-IV) (Hodgins 2004). A score from 5 to 10 indicates a likely diagnosis of pathological gambling per the DSM-IV, 3–4 indicates moderate but subclinical gambling problems, 1–2 indicates a mild but subclinical risk for gambling problems and 0 indicates no problematic gambling. The NODS has been used in previous Swedish treatment studies on problem gambling (Carlbring et al. 2010; Carlbring and Smit 2008). For the purpose of the study, the NODS was modified to assess the last month instead of the last year, and the scale was administered to determine the measures at treatment start, post-treatment and at follow-up. Only the gambler filled out the NODS.

The Time-Line Follow-Back for Gambling (TLFB-G) (Weinstock et al. 2004) was used to report the net losses on gambling in the last month, in accordance with recommendations in the Banff Statement (Walker et al. 2006) that specifies that net losses and time spent on gambling are the most important aspects of a change in gambling behavior. The TLFB-G was administered to determine the measures at treatment start, weekly during treatment, post-treatment and at follow-up. Both the gambler and the CSO estimated the gambler’s gambling by filling out the TLFB-G.

### Secondary Outcomes

Secondary outcomes included measures on anxiety, depression (all gamblers and CSOs), alcohol consumption, and the experience of the treatment for the gamblers and for those CSOs who were included in the BCT arm. Depression was measured using the Patient Health Questionnaire (PHQ-9) (Kroenke et al. 2010) and anxiety was measured using the General Anxiety Disorder 7-item (GAD-7) scale (Spitzer et al. 2006). Alcohol consumption, and consequences of alcohol consumption, was measured using the Alcohol Use Disorders Identification Test (AUDIT) (Saunders et al. 1993). The PHQ-9 consists of nine items scored from 0 to 3 with a total score of 27. A score from 20 to 27 corresponds to severe major depression, 15–19 to moderately severe major depression, 10–14 to minor depression, 5–9 to minimal symptoms and 0–4 to no depression. GAD-7 consists of seven items scored from 0 to 3, with a total score of 21: 15–21 corresponds to severe anxiety, 10–14 to moderate anxiety, 5–9 to mild anxiety and 0–4 to no anxiety. AUDIT contains of 10 items scored from 0 to 4, with a total score of 40. A score above 7 for men and above 5 for women indicates hazardous or harmful use.

The PHQ-9 and GAD-7 were administered to determine the measures before treatment start, at post-treatment and at follow-up. AUDIT was administered before treatment. Participants were also asked free-form text questions regarding how they experienced the treatment, the platform and their assigned therapist. They also ranked the program from 1 to 5 where 1 means “dissatisfied” and 5 means “satisfied”.

## Treatment Arms

After randomization, the participants gained access to a treatment website containing their respective treatment programs. The BCT as well as the CBT program consisted of one chapter/module each week, containing text material, short films and tasks related to a specific treatment component. Each module was, on average, 5–10-pages long. In the BCT condition, the gambler and the CSO were given 10 modules each. In the CBT condition, the gambler was given 10 modules but the CSO was not given any modules. The content in the treatment modules for the gamblers was constructed to be as similar as possible, regardless of the trial arm. Each treatment arm contained 10 modules, thus lasting 10 weeks. Participants were, however, given the opportunity to complete the program during a 12-week time frame to increase flexibility. The modules were made available to the participants one at a time as participants advanced in the treatment.

Although the CBT arm and the BCT arm for the involved gamblers were similar to a large degree, the gamblers in the BCT condition were asked to collaborate with their CSO throughout treatment and several exercises in each module were designed to involve both the gambler and the CSO.

The modules were complemented with scheduled telephone and e-mail support from their assigned therapist. E-mail communication was administered via an online messaging system that is built into the treatment platform. The gamblers and the CSOs had their own log-ins and were not able to read each other’s content or communications.

## The CBT Condition (Gamblers Only)

The content in the CBT condition was based on existing CBT treatments for gambling (Gooding and Tarrier 2009), including cognitive strategies for handling gambling cognitions and cravings, and behavioral strategies such as behavioral activation and functional analysis to identify and manage gambling triggers and reinforcers of gambling behavior. It also included exercises aimed at motivation enhancement and psychoeducation about problem gambling. The treatment is largely based on manuals from a previous study on an Internet-delivered CBT treatment for problem gambling in Sweden (Carlbring and Smit 2008), as well as on a Swedish CBT manual for treating problem gamblers (Ortiz 2006). Some components regarding communication training were included in the treatment so as to be as similar to the BCT gambler condition as possible.

## BCT for the Gambler

The BCT condition was inspired by existing BCT treatments for alcohol and substance use (O’Farrell and Fals-Stewart 2006). Since BCT has never been tested for problem gambling, our treatment was modified to suit problem gambling. Most parts of the original BCT manual mirror components in CBT manuals for problem gambling such as functional analysis and behavioral activation. However, some gambling-specific components such as strategies for handling gambling cognitions and psychoeducation about gambling were taken from the above-mentioned Swedish manuals on CBT treatment for problem gambling (Carlbring and Smit 2008). In general, there was little difference for the gambler between the CBT condition and the BCT condition, except for the added involvement of a CSO in the BCT condition.

## BCT for the CSO

While the above-mentioned BCT manual (O’Farrell and Fals-Stewart 2006) served as the basis for the CSO condition, there were some changes made to suit the CSOs of problem gamblers. BCT relies quite heavily on functional analysis, requiring the CSO to identify the link between the triggers, abuse and consequences for their addicted partner. One purpose is to reward sober behavior, but since gambling produces no physiological signs, it is virtually impossible for a CSO to know when a person is “sober” from gambling. The CSO modules thus borrowed components regarding gambling from a Swedish CBT-based CSO manual (Nordell 2005) and from an Internet-based study on support for the CSOs of problem gamblers (Magnusson et al. 2015). BCT was developed with married or cohabiting couples in mind, while this study accepted any type of personal relationship, e.g. parent–child, friends or siblings. The modules in this study were constructed to be suitable for any type of CSO, for example by giving a variety of examples and by phrasing examples with “Many CSOs feel…” or “Some CSOs have experienced….”

## Therapists

The study’s therapists were master-level clinical psychology students and experienced staff from the Swedish National Gambling Helpline that have training in motivational interviewing (MI) (Miller and Rollnick 2012). The therapists received training in the study manual and in Internet-delivered therapy prior to treatment start, and they also received bi-weekly supervision by an experienced CBT therapist. The therapists were instructed to counsel the participants, both gamblers and CSOs, for approximately 15 min a week.

### Methods

Typically, the TLFB-G data on dependency-related behaviours such as gambling is characterized by excess zeroes (days with no gambling) and skewed distributions for subjects engaging with their problematic behaviour (Bandyopadhyay et al. 2011). Therefore, to properly model the gambling behaviour, we used a two-part semicontinuous model, with a Bernoulli part modelling whether subjects gambled or stayed abstinent, and a gamma distribution to model the money lost when they gambled (Olsen and Schafer 2001). Correlated random intercepts were included for both parts of model.

Since we administered the TLFB-G every week during the treatment period, we chose to aggregate all TLFB-G into average daily losses per week. This ensures that zero values had the same interpretation for all time points. Time was modeled by including week as a continuous variable, we allowed for quadratic change by modeling time using a restricted cubic spline. Since the sample size is small we used three knots, placed at the 10^th,^ 50th, and 90^th^ percentile, to avoid overfitting (Harrell 2015). Moreover, since most participants stopped gambling once they entered treatment, we allowed for discontinuous change by including a dummy variable that separated the baseline from post-baseline measurements (Singer and Willett 2003). To adjust for baseline responses we constrained the baseline measures to be the same for both the groups (Dinh and Yang 2011; Fitzmaurice et al. 2012).

For the other self-report measures—the NODS, PHQ-9 and GAD-7—we used ordinary linear mixed models (Hesser 2015). The time variable was modeled as a categorical variable with subject-specific varying intercepts.

### Bayesian Estimation

We used Bayesian methods to calculate the point estimates and uncertainty intervals of the treatment effects. The Bayesian Markov chain Monte Carlo (MCMC) method is well suited for quantifying uncertainty in semi-continuous models (Neelon et al. 2016), as well as with small sample sizes and with many zeroes (Ghosh et al. 2006). Just like the maximum likelihood estimation (MLE), Bayesian MCMC allows all the available information to be included in the analysis, thus yielding correct inferences under the missing at random (MAR) assumption. For all models, weakly informative priors were used for the variance parameters and non-informative priors for the fixed effects parameters.

We summarized the results as posterior medians and 95% credible intervals (CIs). We calculated the overall money lost by averaging over the two-parts of the model, using the full posterior distribution. Since, we did not average over the random effects, these results are interpreted as the average daily losses per week for individuals with a median amount of losses. To ensure that our model was correctly coded and to evaluate its frequentist properties, we ran a Monte Carlo simulation that showed that our 95% CIs had nominal coverage rates (Smith et al. 2014).

### Posterior Predictive Checks

We assessed the model’s ability to capture key quantities of the observed data via posterior predictive checks (Gelman et al. 1996). This is performed by simulating new data sets from the posterior predictive distribution and then comparing these replicates to the observed data. If the model described the data, well-simulated data sets should capture important features of the data. We also checked how simpler models than the longitudinal semi-continuous model fit the data.

## Results

### Descriptive Statistics of the Participants

Table 1 shows the descriptive demographic statistics of the included participants at baseline. As is evident in Fig. 1, however, a number of participants (“No contact”) were excluded prior to randomization since we could not get in touch with them. Table 2 shows the descriptive statistics for this group compared to the randomized group with regards to the baseline measures. *P*-values are calculated using the independent samples *t* test.

**Table 1:** Descriptive statistics for the included participants

	Mean Age	Gender Female/Male	Mean years of problem	Median gambling debt	Most problematic games	Earlier attempts to quit
Gamblers (all)	36.8	2/16	6.2	300,000 SEK (≈33,253 USD)	Internet casino: 8 Internet betting: 5 Internet poker: 2 Live casino: 1 Bookmaker betting: 1 Trading: 1	Yes: 17 No: 1
Gambler CBT	33.9	0/8	5	115,000 SEK (≈12,747 USD)	Internet casino: 2 Internet betting: 2 Internet poker: 2 Bookmaker betting: 1 Trading: 1	Yes: 7 No: 1
Gambler BCT	39.1	2/8	7.2	415,000 SEK (≈46,000 USD)	Internet casino: 6 Internet betting: 3 Live casino: 1	Yes: 10 No: 0
CSO (all)	41.9	16/2	6.3	–	–	–
CSO CBT	44.6	9/1	6.4	–	–	–
CSO BCT	39.7	7/1	5.7	–	–	–

**Table 2:** Descriptive statistics for the included participants compared to the participants we failed to reach (“No contact”) as measured prior to randomization

	Randomized gamblers (N = 18)		No-contact gamblers (N = 9)		*P*-value	Randomized CSOs (N = 18)		No-contact CSOs (N = 6)		*P*-value
	Score	SD	Score	SD		Score	SD	Score	SD	
NODS	6.6	1.4	7.4	1.2	.124	–	–	–	–	–
PGSI	19.1	4.2	23.4	2.7	.009*	–	–	–	–	–
PHQ-9	11.6	6.5	15.2	6.5	.186	8.7	6.9	11.0	7.5	.487
GAD-7	8.2	5.0	12.0	5.1	.080	8.6	5.9	10.0	5.7	.608
AUDIT	4.2	3.0	8.0	6.4	.045*	3.2	2.8	6.3	6.0	.093

* Significant at the 0.05 level

### Primary Outcome: TLFB-G, Money Lost

Gamblers in both groups showed large reductions in money lost to gambling early in treatment, as measured by the TLFB-G. However, there were no significant differences between the groups.

**Fig. 2:**
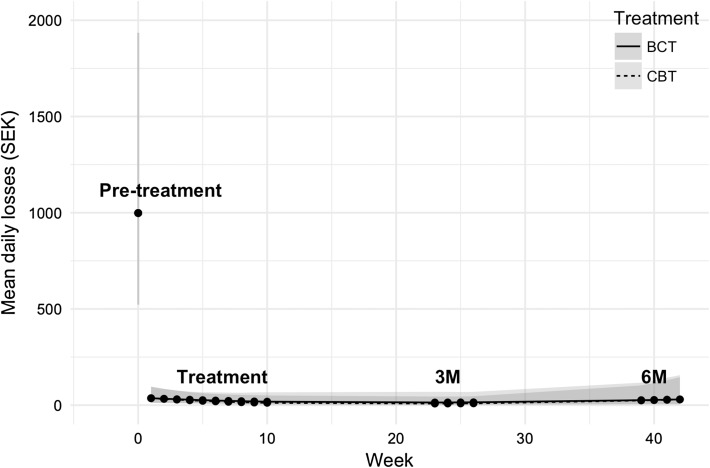
Money lost on gambling

Figure 2 shows the predicted values for the median amount of money lost on gambling per day, as rated by the gambler. The BCT gambler is modeled to have lost 999 SEK (≈111 USD) 95% CI [524, 1935] per day at pre-treatment. This amount changed by −977 SEK (≈−108 USD) 95% CI [−1906, −511] per day at post-treatment, by −980 (≈−109 USD) at 3-month follow-up 95% CI [−1907, −514], and by −957 (≈−106 USD) 95% CI [−1907, −514] at 6-month follow-up. In comparison, the CBT group displayed a similar path. The CBT group is modelled to have lost 5 SEK less than the BCT (≈−0.59 USD) at post-treatment 95% CI [−35, 45], −4.7 SEK (≈−0.52 USD) 95% CI [−35, 52] at 3-month follow-up and −0.67 SEK (≈−0.07 USD) 95% CI [−112, 118] at 6-month follow-up.

Posterior predictive checks did not show any large discrepancies between the statistical model and the observed data for the above-presented TLFB-G results. Key quantities such as the proportion of zeros, the median and maximum amount of money lost, and the observed variances were well captured by the model.

### Primary Outcome: NODS

**Fig. 3:**
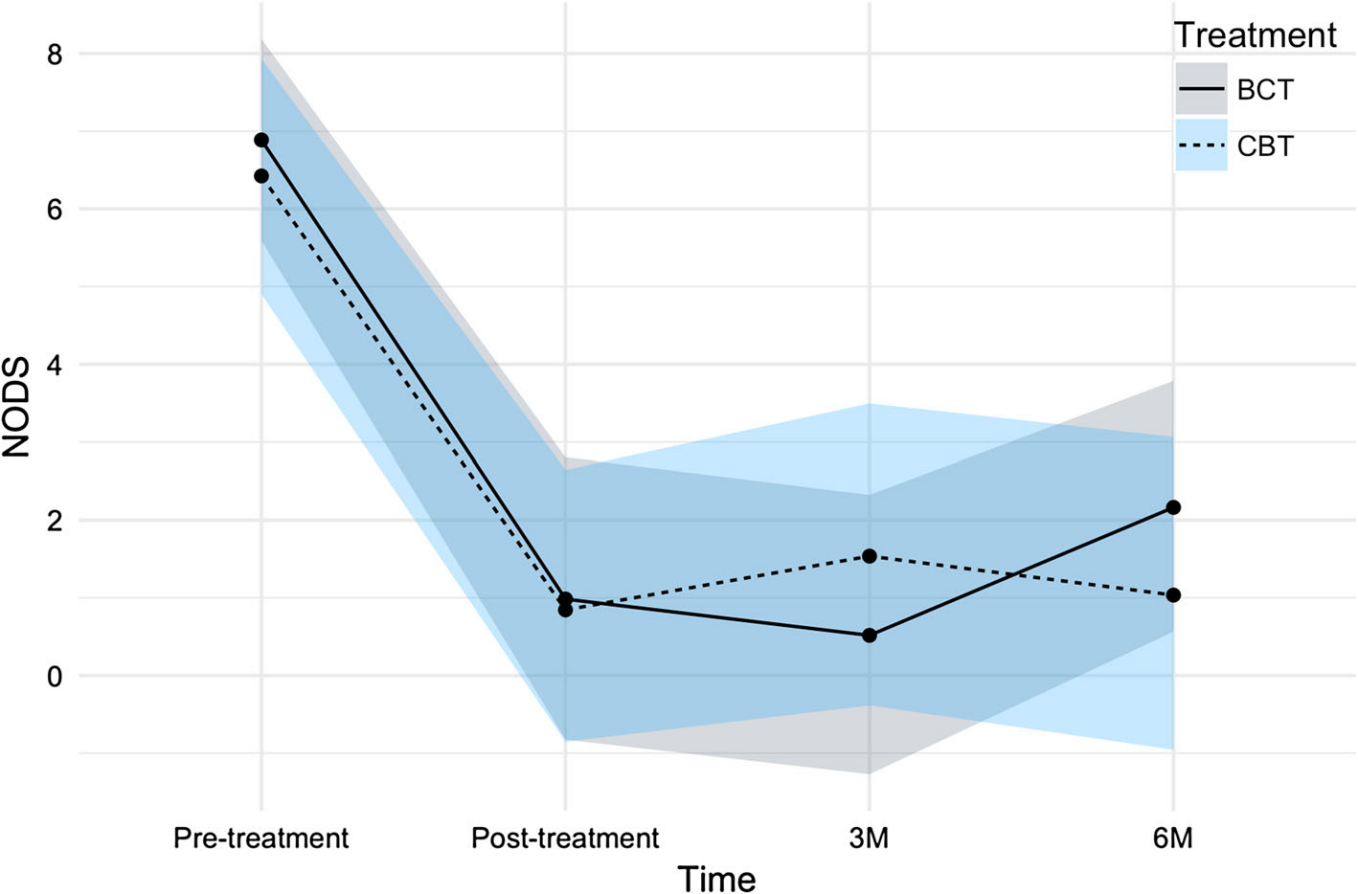
NODS results

Figure 3 shows the changes in the NODS scores measured at four time points. The BCT group changed from 6.89 at pre-treatment to 2.17 at the 6-month follow-up with a 95% CI [−6.7, −2.78], while the CBT group changed from 6.43 at pre-treatment to 1.5 at the 6-month follow-up. At the 6-month follow-up, the two groups differed by 0.67 points on the NODS with a 95% CI [−3.77, 2.46].

### Secondary Outcomes: PHQ-9, GAD-7 for Gamblers

Gamblers in both groups improved their scores considerably on GAD-7 and the PHQ-9. For the PHQ-9, both groups followed a very similar pattern, with a sharp reduction from a baseline score corresponding to moderate depression to post-treatment with a score corresponding to mild depression, followed by a slight increase at the 3-month follow-up. There was further improvement for the CBT group gamblers, reaching a score corresponding to no depression at the 6-month follow-up, which was not seen in the BCT group (see Table 3 for details).

**Table 3:** Gambler scores on secondary outcomes for the PHQ-9 and GAD-7

	BCT gambler		CBT gambler	
	Score	95% CI	Diff. from BCT	95% CI
Patient Health Questionnaire (PHQ-9)				
Pre-treatment	11.68	[7.32, 16.16]	0.58	[–6.23, 7.72]
Post-treatment change	–6.44	[–11.04, –1.64]	–0.76	[–7.25, 5.75]
3-month change	–5.17	[–10.15, 0.14]	0.13	[–7.36, 7.36]
6-month change	–5.03	[–9.66, –0.30]	–2.99	[–10.18, 3.87]
Generalized Anxiety Disorder (GAD-7)				
Pre-treatment	8.19	[5.13, 11.32]	0.61	[–4.27, 5.63]
Post-treatment change	–4.31	[–8.06, –0.51]	–2.37	[–7.79, 3.10]
3-month change	–5.30	[–9.53, –1.22]	0.76	[–5.24, 6.84]
6-month change	–2.55	[–9.09, 2.48]	–3.48	[–9.09, 2.48]

For GAD-7, there was a sharp decrease in symptoms for both groups post-treatment, with greater reductions in the CBT group. At the 3-month follow-up, both groups had a similar score, but at the 6-month follow-up, the groups had slightly different scores, as the CBT group decreased their symptoms and the BCT group increased their symptoms. Scores for both groups corresponded to mild anxiety for all measurements, except for the 6-month follow-up for the CBT group corresponding to no anxiety (see Table 3 for further details).

### Secondary Outcomes: PHQ-9, GAD-7 for CSO

The CSOs in both groups did not have equal scores on the PHQ-9 and GAD-7 at treatment start, and while the BCT group’s CSOs went from moderate depression to no depression and from moderate anxiety to no anxiety, the CBT group’s CSOs did not improve. The CBT group’s CSOs did not change from baseline to post-treatment, but deteriorated sharply at the 3-month follow-up, followed by a recovery to earlier levels at the 6-month follow-up (see Table 4 for details).

**Table 4:** The CSO scores on secondary outcomes for the PHQ-9 and GAD-7

	BCT CSO		CBT CSO	
	Score	95% CI	Diff. from BCT	95% CI
Patient Health Questionnaire (PHQ-9)				
Pre-treatment	10.17	[5.87, 14.29]	–3.33	[–9.34, 3.07]
Post-treatment change	–5.45	[–9.94, –1.02]	3.63	[–3.29, 10.28]
3-month change	–6.97	[–11.77, –2.31]	10.36	[3.27, 17.62]
6-month change	–6.07	[–10.80, –1.33]	4.62	[–2.66, 11.68]
Generalized Anxiety Disorder (GAD-7)				
Pre-treatment	10.28	[6.88, 13.58]	–3.87	[–8.82, 1.53]
Post-treatment change	–5.61	[–9.21, –1.98]	4.30	[–1.28, 9.66]
3-month change	–6.19	[–10.24, –2.45]	8.32	[2.28, 14.05]
6-month change	–6.59	[–10.43, –2.82]	5.70	[0.00, 11.44]

## Feedback from Participants

All participants were asked to rate their experience of the treatment by answering the following questions post-treatment (see also Table 5):

**Table 5:** Feedback from participants

	BCT gambler (N = 5)	CBT gambler (N = 6)	BCT CSO (N = 9)	CBT CSO (N = 7)
Program satisfaction (1–5):	4.8	4.3	4.8	3.3
Would you recommend the program?	Yes: 5, No: 0	Yes: 6, No: 0	Yes: 8, No: 0	Yes: 6, No: 1
Most satisfied with:	Content CSO involvement	Content Therapist Accessibility	Content Support for the gambler Working with gambler	Gambler receiving treatment
Least satisfied with:	Nothing The Internet forum	Too little Therapist Own effort	Too little	Not receiving own modules

- How satisfied are you with “Gambling Free Together” on a scale from to 15 (1 = dissatisfied, 5 = satisfied)?
- Would you recommend “Gambling Free Together” to other persons who are in a similar type of situation as you are?
- The aspects of “Gambling Free Together” I am most/least satisfied with are…?

The questions regarding what the participants were the most and least satisfied with were given with the option to answer with free text. The most common replies are shown in Table 5. Answers containing specific components of the treatment are called *content,* while anything related to the feedback received from the therapist (e.g. telephone calls, e-mail support) is called *therapist*.

## Feedback from the Therapists

The two therapists were asked general questions on their experience of working on this pilot study. While an exhaustive account of their experiences is beyond the scope of this article, a brief résumé of their answers provides an insight into the feasibility of conducting a full-scale RCT. The questions were as follows:

- What is your general experience of working with “Gambling Free Together”?
- What would you change in “Gambling Free Together” if you had the opportunity?
- What were the greatest challenges in working with “Gambling Free Together”?
- What was most time-consuming when working with “Gambling Free Together”?

The therapists generally emphasized the positive aspects of the study: the length of the treatment, the content in the modules and the satisfaction of working with participants who benefited from the interventions.

In terms of the negative aspects, both therapists stressed that they spent a lot of time trying to reach participants who did not answer their e-mails or telephone calls. According to the therapists this could be attributed to a lack of motivation on behalf of the participants, but perhaps also to the participants’ chaotic life circumstances. One therapist described this chasing as “tiresome and demoralizing.” In many cases, the two participants in the BCT condition would complete the modules at a very different pace. This posed a problem to the therapists, but also to the treatment program itself, since the program builds on the assumption that participants will cooperate and perform certain exercises together. One therapist suggested that some exercises concerning relationship functioning should be provided earlier in the treatment program to give the participants “a common ground and a common start.”

One therapist asked for more tailored interventions that could be adapted to suit the different needs of the participants. Both therapists concluded that a challenge to this intervention was when the CSO was more motivated than the gambler and could be assumed to have pushed the gambler into treatment.

## Discussion

The aim of this study was two-fold: To study the possible differences between BCT and CBT for problem gambling and to investigate the feasibility of an Internet-based relationship intervention for problem gambling.

## Treatment Results

Both treatments successfully lowered the symptoms of problem gambling and measures of depression and anxiety for the gamblers. Both groups went from NODS levels corresponding to pathological gambling to levels corresponding to a mild but subclinical risk for problem gambling. The NODS findings were consistent with results from the TLFB-G indicating rapid decreases in time spent and money lost on gambling after the intervention had started. There were no clear differences between the two conditions on any gambling-related outcome measure, since both treatments significantly lowered all outcomes related to gambling. Most gamblers in both groups seemed to abstain from gambling altogether during the treatment period, but it is unclear if this represents a permanent change in gambling behavior. This makes it difficult to draw conclusions regarding the relative efficacy of the treatments.

Using the TLFB-G to measure gambling behavior every day provides us with the opportunity to closely monitor the progress of the participants. The amount wagered seemed to be rather unevenly distributed, where short episodes of “binge gambling” were succeeded by longer periods of complete abstinence. During a binge episode, gamblers often spent large sums of money, while little or no money was spent during periods of abstinence. This poses a challenge in terms of how the data should be analyzed statistically, but also what it means clinically. It is not clear at what level of spending participants could be classified as recovered, and whether a sharp decrease or increase in spending is mirrored by a similar change in problem gambling symptoms overall. Put differently, is 5000 SEK lost on gambling five times worse than 1000 SEK lost on gambling in regards to harm and the degree of addiction? There are also many different ways in which to measure the TLFB-G: either as money lost per day, as number of days gambled, as a proportion of days gambled or as money lost on gambling days. This relates to an overarching issue on how we define recovery and harm in association with gambling and what it means for problem gambling. When has someone recovered and when does gambling become harmful? While it is possible to provide a general answer to these questions such as cut-off scores on NODS or number of DSM-5 criteria, it is a challenge to pinpoint the exact thresholds for recovery and harm when it comes to its relation to time or money spent on gambling.

As for many other studies on interventions for problem gambling (Melville et al. 2007), attrition was high, especially among the gamblers. Since this was merely a pilot study, the initial number of participants was already small, making it even harder to draw conclusions, as is illustrated by some of the large CIs. It is difficult to know whether those who dropped out differed in any substantial way from the completers. They could possibly have had more ongoing gambling problems, but they could also have felt that they did not need treatment any longer. Comparisons between included participants and prospective participants that signed up but never initiated treatment indicate that there were differences between these two groups. The latter group had significantly worse gambling problems and had a higher score on AUDIT; a measure of alcohol consumption and harm from alcohol use. This could indicate that this type of treatment appeals less to gamblers with more complex problems. The study’s design, where gamblers and CSOs are recruited pairwise, is modeled to increase gambler retention in treatment, given results from earlier studies identifying CSO involvement as positive for treatment participation and retention. However, there may also be a risk that unmotivated gamblers are pressured into treatment by their CSO, which could possibly make the gambler even less motivated in terms of participating in treatment.

Measures for anxiety and distress indicate that gamblers in both groups, as well as the CSOs in the BCT group, improved during the course of treatment. The CSOs in the CBT group, however, did not seem to improve substantially during the course of treatment. This seems logical since they did not receive any intervention, and could provide indirect support for the possible benefit of CSO directed treatment. However, this also indicates that the CSOs do not necessarily benefit solely from improvements in the gambler’s gambling behavior. Given that the main reason for seeking treatment is to target the gambling problems of the gambler, this may either be regarded as somewhat surprising, or as an indication of the severe negative effects that gambling may exert on significant others. While the exact reasons for these results remain to be uncovered, one interpretation is that the CSOs of problem gamblers need interventions tailored specifically for them, regardless of how the gambling problem develops. One reason for this could be that CSO-tailored interventions give insights into how problem gambling develops, how it is maintained and how it can be overcome. Since many problem gamblers have a history of alternating periods of abstinence and relapse, the CSOs may not feel sufficiently reassured by recent changes in gambling behavior alone.

## Feasibility and Acceptability

The participants receiving treatment generally rated it very favorably, especially those who received BCT. While all BCT gamblers but one gave the treatment the highest rating, the CBT gamblers had a slightly more mixed experience. A few of them mentioned their own lack of self-discipline as a reason for not being completely satisfied with the program. This could be interpreted as an advantage for the BCT condition, where CSO involvement could encourage treatment engagement. The CSOs who did not receive any treatment were generally less positive and some of them expressed disappointment at not receiving any modules or therapist support. However, as with any intervention affected by attrition, and the fact that this is a limited pilot study, these results should be interpreted with caution. Participants who were not satisfied with the treatment program could also be less motivated to complete the treatment and to fill out the treatment evaluation form.

The therapists were also generally positive about the study design regarding involving both the gambler and the CSO in the treatment. However, they both concluded that a substantial amount of their time was used up in reminding patients to complete modules and in arranging and re-arranging scheduled telephone calls. They also remarked on how some of the CSOs seemed considerably more motivated to participate than the gambler did, which became obvious in the screening process. This posed a particular challenge when they were randomized to the CBT condition where the CSOs did not receive any treatment. Some of these issues relate to the feasibility of conducting Internet-based interventions targeting more than one participant rather than to the feasibility of interventions targeting gambling.

## Conclusions

Several questions remain to be answered regarding the outcomes of Internet-delivered BCT treatment for problem gambling. Long-term results and an increased power could allow for more in-depth analyses regarding the efficacy of BCT for gambling. Understanding why participants drop out will also be essential in terms of evaluating the potential of BCT treatment. A full-scale RCT is underway (Nilsson et al. 2016) that will hopefully give clarity to some of these issues. Given the high approval ratings by the participants, as well as the substantial reductions in gambling by the participants, this pilot study has shown that it is reasonable to further investigate the potential of BCT in a full-scale RCT.
